# Nanowire arrays restore vision in blind mice

**DOI:** 10.1038/s41467-018-03212-0

**Published:** 2018-03-06

**Authors:** Jing Tang, Nan Qin, Yan Chong, Yupu Diao, Yiliguma, Zhexuan Wang, Tian Xue, Min Jiang, Jiayi Zhang, Gengfeng Zheng

**Affiliations:** 1 Laboratory of Advanced Materials, Institutes of Brain Science, State Key Laboratory of Medical Neurobiology, Shanghai Key Laboratory of Molecular Catalysis and Innovative Materials, Department of Ophthalmology, Zhongshan Hospital, Fudan University, Shanghai 200032, China; 2 School of Life Sciences and Hefei National Laboratory for Physical Sciences at Microscale, University of Science and Technology of China, Hefei 230026, China

## Abstract

The restoration of light response with complex spatiotemporal features in retinal degenerative diseases towards retinal prosthesis has proven to be a considerable challenge over the past decades. Herein, inspired by the structure and function of photoreceptors in retinas, we develop artificial photoreceptors based on gold nanoparticle-decorated titania nanowire arrays, for restoration of visual responses in the blind mice with degenerated photoreceptors. Green, blue and near UV light responses in the retinal ganglion cells (RGCs) are restored with a spatial resolution better than 100 µm. ON responses in RGCs are blocked by glutamatergic antagonists, suggesting functional preservation of the remaining retinal circuits. Moreover, neurons in the primary visual cortex respond to light after subretinal implant of nanowire arrays. Improvement in pupillary light reflex suggests the behavioral recovery of light sensitivity. Our study will shed light on the development of a new generation of optoelectronic toolkits for subretinal prosthetic devices.

## Abstract (web summary)

The restoration of light response using retinal prosthesis could be a way to restore vision following retinal degenerative disease. Here the authors develop gold-titania nanowire arrays that restore visual response in blind mice.

## Introduction

Retina is an important light-sensitive tissue that transduces light information into neural activities through multi-layers of neuronal cells^1–5^. Light entering an eye passes through the transparent retina and is mostly captured by the visual pigment-containing photoreceptors^6–9^. Retinal degenerative diseases such as retinitis pigmentosa and macular degeneration lead to irreversible damage or even loss of photoreceptors, which can result in serious impairment of vision and eventually blindness^10,11^. The state-of-art retinal prosthesis devices require the use of a subretinally placed photodiode array detecting near-infrared signals from a video capturing camera^12,13^. Recently, developing light-responsive materials as artificial photoreceptors for interfacing with blind retinas has emerged as a promising alternative for retinal prosthesis, with several exciting preliminary demonstrations using metal electrode arrays^14^, cadmium sulfide-carbon nanotubes^15^, semiconductor silicon photodiodes^16^ or conducting polymers^17–19^. However, these photoresponsive devices require additional microelectronic processing for signal generation, transduction and processing, posting limitations for in vivo applications. Breaking this bottleneck requires capabilities for engineering large interaction surfaces/interfaces between retinal cells and semiconductor micro/nanostructures. Recently, subretinal implantation of photovoltaic polymers restores light sensitivity in blind rats^19^. However, spatiotemporal characterizations of retinal responses are not clear.

**Fig. 1:**
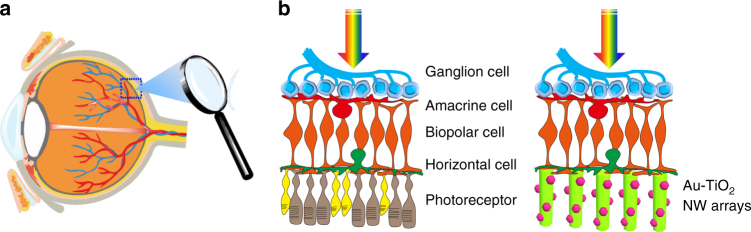
Retina-nanowire interfaces. **a** Illustration of an eye. **b** Comparison of a retina and NW arrays-interfaced blind retina that lacks photoreceptors. The necrotic photoreceptor layer (rod and cone cells) in the blind retina is replaced by an Au–TiO_2_ NW array as artificial photoreceptors

Among the potential candidates of photoresponsive materials targeting the goal of artificial photoreceptors, the ordered, oriented one-dimensional (1D) semiconductor nanowire (NW) arrays exhibit high surface areas, large charge transport mobility, excellent biocompatibility and stability^20–22^. Distinctive from previous reports of photoresponsive materials/structures, the high orientation and anisotropy of 1D NW arrays are analogous to the morphology and architecture of photoreceptors (Fig. 1a, b), and thereby enable efficient photoabsorption and charge separation that are similar in photoconversion devices such as solar cells^23^ or photodetectors^24^. The oriented semiconductor NW arrays are capable of generating photocurrent upon light illumination to depolarize neurons^25^. In addition, as no trans-ocular cables or power supplies are needed, a high and uniform areal density of photoresponsive units is expected for optimized spatial resolution. More uniquely, the surface roughness of oriented NW arrays may further enhance interfacing efficiency with innate retinal circuits including bipolar cells, which is crucial for the artificial retinal prosthesis towards visual function restoration.

Herein, we demonstrate an oriented gold nanoparticle-decorated titania (Au-TiO_2_) NW arrays-based artificial photoreceptor interfaced with retinal degenerated 1 (*rd1*) /cone diphtheria toxin subunit-A (*cDTA*) blind mouse retinas, for real-time transduction of photo-coded information. Retinal ON responses to green, blue and near UV light are robustly recorded with high spatial and temporal resolutions, indicating direct stimulation of bipolar cells without affecting the rest of retinal circuit. More excitingly, with NW arrays subretinally implanted into blind mice, light-evoked activities in the primary visual cortex (V1) in vivo, as well as improved pupillary light reflex (PLR) in awake-behaving mice confirm the recovery of visual function 4–8 weeks after implant surgery, indicating the potential of using NW arrays as subretinal prosthetic devices.

## Results

### Fabrication of Au nanoparticle-decorated TiO_2_ NW arrays

**Fig. 2:**
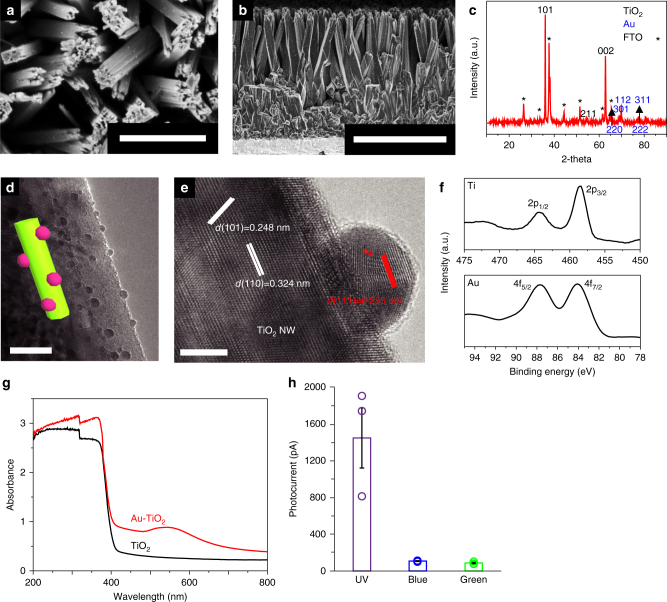
Photovoltaic performances of Au-TiO_2_ NW arrays. **a** Top-view and **b** side-view scanning electron Mmcroscope images of Au–TiO_2_ NW arrays. **c** XRD spectra collected for Au–TiO_2_ NW arrays. The black and blue numbers indicate the diffraction peaks of TiO_2_ and Au, respectively. Diffraction peaks of the FTO substrate are marked by asterisks. **d** TEM and **e** HRTEM images of a representative Au–TiO_2_ NW, where Au nanoparticles were grown on the NW surface. Inset in **d**: schematic of the Au–TiO_2_ NW structure. **f** XPS spectra of Au–TiO_2_ NW arrays. Top: Ti 2p_1/2_, and 2p_3/2_ peaks. Down: Au 4f_5/2_ and 4f_7/2_ peaks. **g** UV-visible absorbance spectra of TiO_2_ and Au-TiO_2_ NW arrays on FTO substrates. **h** Histogram of photocurrents from Au–TiO_2_ NW arrays by illumination of UV, blue and green light, respectively (*n* = 3). Different filters were applied to obtain near UV (375/28 nm, full intensity 133 μW mm^–2^), blue (470/20 nm, full intensity 691 μW mm^–2^), and green light (546/12 nm, full intensity 470 μW mm^–2^). Data are presented as mean and standard error of mean (S.E.M.). Scale bars: 500 nm (**a**), 2 μm (**b**), 20 nm (**d**), 5 nm (**e**)

The oriented Au–TiO_2_ NW arrays were grown on fluorine-doped tin oxide (FTO, Supplementary Fig. 1) or flexible polymer substrates (Supplementary Fig. 2) by a hydrothermal method^26,27^, followed by in-situ reduction of gold salt on the surface of pre-formed TiO_2_ NW arrays (Methods). The obtained Au-TiO_2_ NW arrays had a perpendicular orientation to the underlying substrate, and the average NW diameter and length were ~100 nm and ~2 μm, respectively (Fig. 2a, b), similar to those of pristine TiO_2_ NW arrays before deposition of Au nanoparticles (Supplementary Fig. 1a, b). The density of NWs was ~10 per μm^2^, corresponding to ~10^9^ NW arrays per cm^2^. X-ray diffraction (XRD) patterns of the Au-TiO_2_ NW arrays displayed characteristic reflection peaks of a rutile TiO_2_ crystalline structure (Joint Committee on Powder Diffraction Standards, JCPDS No. 21-1276) and a face-center-cubic Au structure (JCPDS No. 04-0784) (Fig. 2c)^28^. Transmission electron microscopy (TEM) images showed the average size of surface-decorated Au nanoparticles was ~10 ± 5 nm, with the inter-particle distance ranging from 10 to 30 nm (Fig. 2d). High-resolution TEM (HRTEM) images (Fig. 2e, Supplementary Fig. 1c) and the selected-area electron diffraction (SAED) patterns (inset in Supplementary Fig. 1c) revealed the single-crystalline nature of both the NW trunks and the surface-decorated Au nanoparticles, respectively. The well-resolved lattice fringes of 0.324 and 0.248 nm (Fig. 2e) were correlated to the *d*-spacing values of (110) and (101) planes of rutile TiO_2_ crystals^29^. The lattice spacing of 0.235 nm was assigned to the (111) planes of Au crystal with a face-center-cubic structure. Energy-dispersive X-ray (EDX) spectroscopy (Supplementary Fig. 3) and mapping of Ti, O and Au (Supplementary Fig. 4) further confirmed the composition of Au-TiO_2_ NW arrays, as well as the uniform distribution of Au nanoparticles over entire TiO_2_ NW arrays. The molar percentages of Au and Ti were calculated as ~0.11% and 71.77%, respectively. X-ray photoelectron spectroscopy (XPS, Fig. 2f) revealed two peaks for Ti as 2p_1/2_ and 2p_3/2_, and two peaks for Au 4f_5/2_ and 4f_7/2_.

The decoration of Au nanoparticles can enhance the photoconversion efficiency of TiO_2_ NW arrays into visible range, due to the electrical field amplification and the injection of surface plasmon resonance generated hot electrons into TiO_2_ conduction band^30^. The photoabsorption property of Au–TiO_2_ NW arrays was first demonstrated by UV-visible absorption spectroscopy (Fig. 2g). Compared to pristine TiO_2_ NW arrays that mainly absorb at the UV region, the Au–TiO_2_ NW arrays show a much enhanced photoabsorption in the visible range, with the peak position centered around 550 nm, in accordance with the surface plasmon absorption of ~10 nm Au nanoparticles^31,32^. The absorption coefficients for Au–TiO_2_ NW arrays were calculated to be ~1.5, 0.4, and 0.5 μm^–1^ in the UV, blue and green regimes, respectively, comparable or better than the absorption coefficients of natural photoreceptors (i.e., 0.02–0.06 μm^–1^ in the green regime)^33^. These enhanced absorption coefficients are beneficial for Au–TiO_2_ NW arrays with a much smaller thickness than normal photoreceptor cell layers, typically on the order of 60 μm.

The photoconversion properties of Au–TiO_2_ NW arrays were then demonstrated by photocurrent measurements under different wavelengths of near UV, blue and green light, respectively (Supplementary Fig. 5, Methods). The typical substrate size was ~1 cm^2^. Compared to the dark state without light, when the Au–TiO_2_ NW arrays were illuminated by different light wavelengths, a clear increase of photocurrent was recorded almost instantaneously, while the shutoff of light led to the drop of photocurrent to the original dark state. The summarized photocurrent response of different light wavelengths show the average magnitudes of photocurrent change were ~1450, 108, and 87 pA, respectively (Fig. 2h).

### Light responses and spatial resolution

**Fig. 3:**
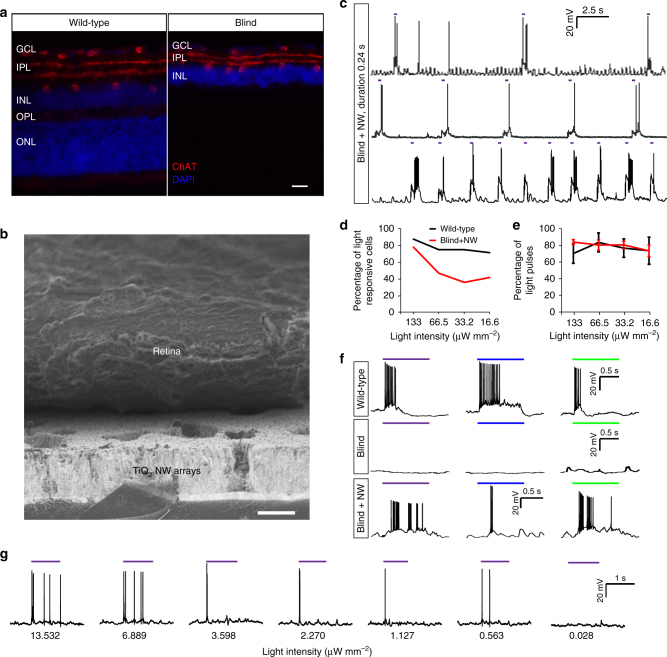
Light responses in NW arrays-interfaced blind mouse retinas. **a** Fluorescent images of retinal slices of wild-type and blind mice immunostaining for ChAT (red) and DAPI (blue). GCL: ganglion cell layer; IPL: inner plexiform layer; INL: inner nuclear layer; OPL: outer plexiform layer; ONL: outer nuclear layer (photoreceptors). **b** SEM images of the interface between the retina and NW arrays. **c** Light responses recorded by whole-cell patch clamp to near UV light stimulation at 0.24 s duration and 10 s (top panel), 5 s (middle panel), 2 s (bottom panel) intervals in NW arrays-interfaced blind retinas. **d** Percentage of light-responsive cells at different light intensities. Wild-type: 133 μW mm^–2^, *n* = 8 RGCs, 6 retinas; 66.5 μW mm^–^^2^, *n* = 8 RGCs, 7 retinas; 33.2 μW mm^–2^, *n* = 8 RGCs, 7 retinas; 16.6 μW mm^–2^, *n* = 7 RGCs, 5 retinas. Blind + NW: 133 μW mm^–2^, *n* = 91 RGCs, 30 retinas; 66.5 μW mm^–2^, *n* = 51 RGCs, 19 retinas; 33.2 μW mm^–2^, *n* = 47 RGCs, 17 retinas; 16.6 μW mm^–2^, *n* = 43 RGCs, 17 retinas (Chi-square test, *P*_133_ = 0.859, *P*_66.5_ = 0.276, *P*_33.2_ = 0.095, *P*_16.6_ = 0.295). **e** Percentage of light pulses triggering spiking activities in all responsive RGCs. Wild-type: 133 μW mm^–2^, *n* = 7 RGCs, 6 retinas; 66.5 μW mm^–2^, *n* = 6 RGCs, 5 retinas; 33.2 μW mm^–2^, *n* = 6 RGCs, 5 retinas; 16.6 μW mm^–2^, *n* = 5 RGCs, 4 retinas. Blind + NW: 133 μW mm^–2^, *n* = 65 RGCs, 25 retinas; 66.5 μW mm^–2^, *n* = 22 RGCs, 17 retinas; 33.2 μW mm^–2^, *n* = 15 RGCs, 11 retinas; 16.6 μW mm^–2^, *n* = 18 RGCs, 11 retinas. (Two-sided Wilcoxon rank-sum test, *P*_133_ = 0.212, *P*_66.5_ = 0.815, *P*_33.2_ = 0.913, *P*_16.6_ = 0.974. Data are presented as mean and S.E.M.) **f** Near UV, blue and green light responses in wild-type, blind and NW arrays-interfaced blind retinas. Horizontal color bars represent the light stimulation color and duration. **g** RGC responses to weak light illumination in NW arrays-interfaced blind retinas. Scale bars: 20 μm (**a**), 5 μm (**b**)

Mice are naturally sensitive to both near UV and visible light. In the following electrophysiology studies, light responses of retinal ganglion cells (RGCs) in wild-type (C57BL/6J) and *rd1*/*cDTA* mouse retinas with NW array interfaces were investigated. Mutations in both *Pde6b* and *cDTA* genes in *rd1*/*cDTA* mice (also referred to as blind mice below) led to complete degeneration of rod and cone photoreceptors by P50 (Fig. 3a, Methods^34^). To eliminate the possibility of some remaining photoreceptors, we conducted more experiments to measure light responses of RGCs in *rd1*/*cDTA* blind retinas. 0/8 RGCs from 4 retinas, 0/7 RGCs from 5 retinas and 0/6 RGCs from 5 retinas responded to near UV (375/28 nm), blue (470/20 nm) and green (546/12 nm) light, respectively, indicating that both rod and cone photoreceptors were completely absent in the *rd1*/*cDTA* blind retina. Au-TiO_2_ NW arrays were then placed underneath the blind retina, with the inner nuclear layer in contact with NW arrays, and the activities of RGCs were recorded using patch clamp pipettes. The retina and NW arrays were in close contact, as shown in scanning electron microscope (SEM) images (Fig. 3b). Contours of individual retinal cells were not visible in SEM images, as the membranes of retinal cells were embedded in the retinal tissue (Supplementary Fig. 6).

It is clear that RGCs in the blind retina robustly responded to near UV light (with a 0.24 s duration and intervals of 10, 5 and 2 s, respectively) (Fig. 3c). In addition, to evaluate the light sensitivity of the NW arrays-interfaced blind retina, the spike activities of RGCs in response to light with different intensities were measured (Supplementary Fig. 7a). The intensity-dependent responses to near UV light were similar or close to those in wild-type mice. The fractions of light responsive RGCs in wild-type mouse retinas were similar for different intensities (Fig. 3d, black curve). In the NW arrays-interfaced blind mouse retinas, the fraction of light responsive RGCs were 78%, 47%, 36% and 42% at 133, 66.5, 33.2, and 16.6 μW mm^–2^, respectively (Fig. 3d, red curve). In addition, RGCs in wild-type mice responded to majority of light pulses (Fig. 3e, black curve). For the NW arrays-interfaced blind mouse retinas, we also showed that the threshold to induce light response was ~0.5 μW mm^-2^ (Fig. 3g**)**, that was 25 lux, which was out of the intensity range of rods (≤0.1 lux). The percentages of pulses that initiated spike activities in RGCs were similar to those of wild-type retinas (Fig. 3e, red curve). The number of spikes for different light intensities, as well as the spike waveforms were also similar (Supplementary Fig. 7b, g). Due to the absence of photoreceptors and therefore sign-preserving and sign-inverting synapses between the photoreceptors and the bipolar cells, only ON responses were recorded in the NW arrays-interfaced blind mouse retinas. The latency of RGCs in NW arrays-interfaced blind retina was similar to those in wild-type retina at different UV light intensities (Supplementary Fig. 7d). The latencies for green and blue light in NW arrays-interfaced blind retina were much larger than those in wild-type retina, most likely due to the fact that the photocurrent induced by green and blue light was smaller than that by UV light.

The sensitivity of different light wavelengths was then investigated. In wild-type mice, RGCs responded to near UV, blue, and green light (with intensities of 133, 691 and 470 μW mm^–2^, respectively) (Fig. 3f, top panel). Without the NW arrays, retinas of blind mice did not respond to either UV, blue or green light (Fig. 3f, middle panel), confirming the blindness of these mice. The representative spiking activities of RGCs in response to near UV, blue and green light were shown in Fig. 3f, bottom panel. For each RGC, at least three light pulses were presented, with approximately 1 min interval between each pulse. The average number of elicited spikes was 11 for near UV, 4 for blue and 6 for green light. Light-responsive RGCs responded to 84% near UV light pulses, 57% blue light pulses and 86% green light pulses, respectively (Supplementary Fig. 7c). Moreover, time evolution of RGC responses were measured (Supplementary Fig. 8), indicating that the interface between NW arrays and retinal cells was purely physical contact and did not evolve over time.

**Fig. 4:**
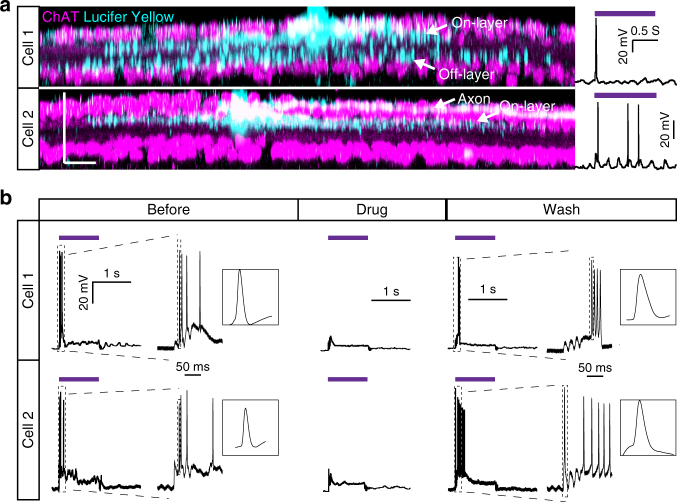
Anatomical and functional On- and Off- features of RGCs in NW arrays-interfaced blind retina. **a** Confocal images showing the distribution of dendritic arbors of two example RGCs (cell 1 and 2, left) and their corresponding near UV light responses (right, 133 μW mm^–2^). White arrows indicate the axon, on-dendritic layer and off-dendritic layer of the Lucifer Yellow labeled RGC. **b** Responses of two example RGCs to near UV light stimulation (133 μW mm^–2^) before, during and after the application of glutamate receptor antagonist. Zoom-in spike waveforms are shown to the right of their original figures. Scale bar: 20 μm (**a**)

In order to examine the anatomical and functional On- and Off-features of RGCs in NW arrays-interfaced blind retina and wild-type retina, we recorded the light responses of RGCs with micropipette filled with internal solutions containing Lucifer Yellow, and then conducted immunostaining of these recorded RGCs to examine the distribution of their dendritic arbors in On and Off layers (labeled by ChAT). We first investigated whether light-sensitive Lucifer Yellow in the pipette affected the light responses in the RGCs^35^. The percentage of responsive RGCs was similar with or without Lucifer Yellow in the pipette (Supplementary Fig. 9d). In the NW arrays-interfaced blind retina, dendrites of some RGCs were located in both On and Off layers (Fig. 4a, top panel). These RGCs exhibited On responses. Some other RGCs had their dendrites distributed in On layer, and exhibited On responses as well (Fig. 4a, bottom panel). These results indicated that RGCs responded to the onset of light by photocurrent from the NW arrays, despite the fact that they received input from both On-center and Off-center bipolar cells. We further examined the light responsive patterns of RGCs in both NW arrays-interfaced blind retina and wild-type retina. It turns out that the ~30% of the RGCs in NW arrays-interfaced blind retina sustained On responses to light stimulation, whereas the rest RGCs showed transient On responses (Supplementary Fig. 7e). This ratio of transient On to sustained On response was similar to that of wild-type RGCs.

To investigate whether the RGC light responses resulted from direct RGC activation or indirect activation of bipolar cells that induced spiking activities in RGCs, synaptic transmission from bipolar cells to RGCs was blocked using glutamate receptor antagonists (Methods). The light responses from RGCs disappeared 4 min after the application of drugs (Fig. 4b, left and middle columns). After washing off the drugs, the light responses were restored (Fig. 4b, right columns**)**. Light responses of all the RGCs were blocked by the drugs, indicating that light responses in RGCs originate mostly from activation of bipolar cells instead of RGCs (Supplementary Fig. 7h).

**Fig. 5:**
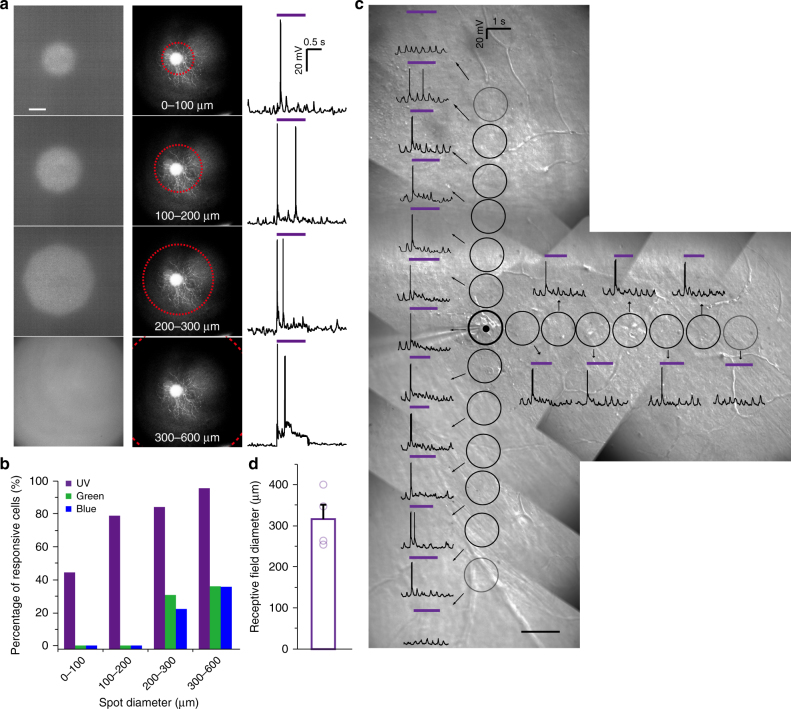
Responses of RGCs to light spots of different sizes in NW arrays-interfaced blind mouse retinas. **a** Left column: near UV light spots with different sizes. Middle column: RGCs (cell bodies and dendritic arborizations) labeled by Lucifer Yellow during whole-cell patch clamp experiments. Right column: responses of RGCs to near UV light spots of different sizes. Purple lines represent the presence of UV light. **b** Fractions of light-responsive RGCs in NW arrays-interfaced blind mouse retinas that responded to UV, green and blue light spots with different sizes (recordings conducted without Lucifer Yellow in the pipette). UV: 0–100 μm, *n* = 18 RGCs, 13 retinas; 100–200 μm, *n* = 19 RGCs, 13 retinas; 200–300 μm, *n* = 19 RGCs, 13 retinas; 300–600 μm, *n* = 23 RGCs, 16 retinas. Green: 0–100 μm, *n* = 13 RGCs, 9 retinas; 100–200 μm, *n* = 12 RGCs, 9 retinas; 200–300 μm, *n* = 13 RGCs, 9 retinas; 300–600 μm, *n* = 25 RGCs, 14 retinas; Blue: 0–100 μm, *n* = 8 RGCs, 5 retinas; 100–200 μm, *n* = 9 RGCs, 6 retinas; 200–300 μm, *n* = 9 RGCs, 6 retinas; 300–600 μm, *n* = 14 RGCs, 8 retinas. **c** A sketch map of receptive field calculation of an example neuron. Black circles represent the different locations of light spot. Black dot represents the location of the neuron. **d** Receptive field diameter of RGCs in NW arrays-interfaced blind mouse retinas. *n* = 4 RGCs in 4 retinas. Data are presented as mean and S.E.M. Scale bars: 50 μm (**a**, **c**)

The spatial resolution of light response in the NW arrays-interfaced blind mouse retinas was further investigated. The photocurrents induced by light spots were measured with different sizes and different distances from the center of the spot (Supplementary Fig. 9a, b). Near UV and green light spots with different diameters were presented to the RGC (Fig. 5a). For blind mouse retinas, the minimum size of light spots to trigger RGC response was 45 µm. When the diameter of near UV light spot was smaller than 100 µm, 44% of RGCs exhibited clear responses (Fig. 5b). The percentages of RGCs that responded to near UV light spots with diameters of 100–200 µm, 200–300 µm, and 300–600 µm were 79%, 84%, and 96%, respectively. Meanwhile, for green and blue light spots with diameters of 200–300 µm, 31% and 22% of RGCs exhibited light responses, respectively; for green and blue light spots with diameters of 300–600 µm, 36% of RGCs exhibited light responses. In Supplementary Figure 9b, the photocurrent increased significantly when the size of the light spots increased from 200 µm to 300–600 µm, which can lead to the increased percentage of responsive RGCs for the light spots of 300–600 µm. For wild-type mouse retinas, RGCs exhibited a typical center-surround light response pattern for both near UV and green light (Supplementary Fig. 9c).

In order to estimate the receptive field size of the light-responsive RGCs, we presented light spots of 45 µm in size to the RGCs (Fig. 5c). Since the light spots were manually moved, the light responses were measured at different distances from the cell body of the RGCs along three axes. The responses were roughly homogenous along the three axes. The average size of the receptive field was 316 µm (Fig. 5d). In addition, we moved the light spot of 45 µm across the RGC (Supplementary Fig. 9e). Localized RGC responses were recorded when the light spot swept around the vicinity of the cell body.

### Population light responses

**Fig. 6:**
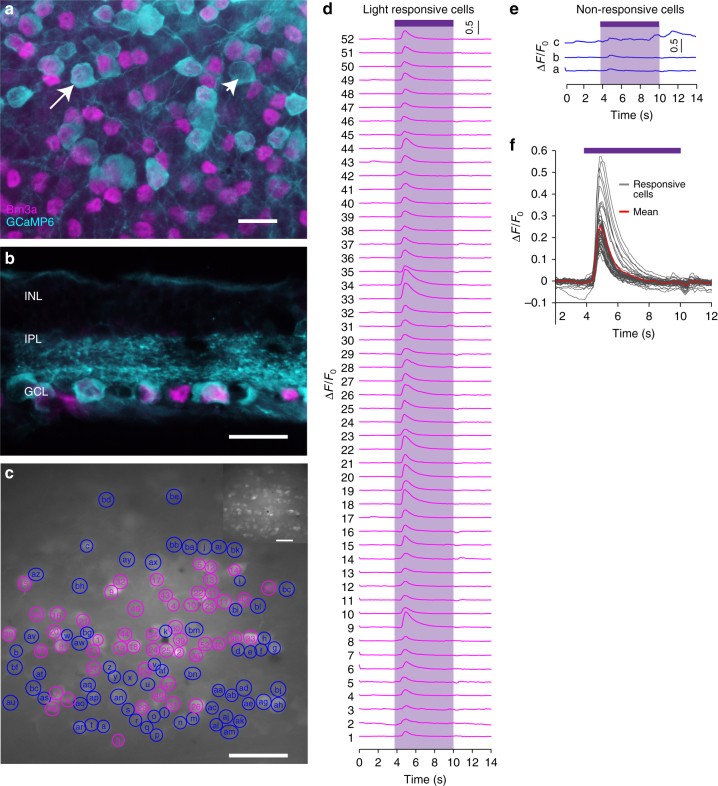
Cellular-level population light responses of RGCs in NW arrays-interfaced blind mouse retinas. **a** Expression of GCaMP6 in the whole-mount retina. Turquoise color represents cell bodies of RGCs that expressed GCaMP6, and magenta color represents Brn3a-labeled RGCs. Arrowhead with long tail: cells co-stained with Brn3a and GCaMP6. Arrowhead with short tail: cells stained with GCaMP6. **b** The cross section of the retina. INL: inner nuclear layer; IPL: inner plexiform layer; GCL: ganglion cell layer. **c** Snapshot image of GCaMP6 fluorescence when no light stimulation was presented. Magenta and blue circles outline 52 light responsive and 66 non-responsive RGCs, respectively. Inset image shows the same snapshot image with no outlines of the RGCs. **d** All the 52 light responsive RGCs in **c**. Purple shade represents the presence of near UV light. **e** Three examples of non-responsive RGCs in **c**. **f** Change of fluorescence (Δ*F*/*F*_0_) for all the responsive RGCs in **c**. Gray lines represent Δ*F*/*F*_0_ curves for individual cells. Red line represents average Δ*F*/*F*_0_ for all the RGCs. Purple lines represent the presence of near UV light. Scale bars: 20 μm (**a**, **b**), 50 μm (**c**)

In order to demonstrate the capability of the NW arrays-based artificial photoreceptors as potential retinal prosthesis materials, the population light responses in the NW array-interfaced blind mouse retinas using calcium-sensitive protein (GCaMP6s) imaging were also illustrated. 88% of GCaMP6s cells expressed Brn3a, a RGC marker, indicating that majority of the GCaMP6s cells were RGCs (Fig. 6a, b, Supplementary Fig. 10). Light induced responses in both RGCs and underlying neuritis (Fig. 6c, d, Supplementary Movie 1 in the Supporting Information). Some cells had no obvious light-evoked responses (Fig. 6e). The light response curves were similar for all the RGCs (Fig. 6f). 46% RGCs had light responses under whole-field illumination, demonstrating global activation of RGCs. Taken together, these results indicate that the NW arrays-interfaced blind mouse retina has good population light responses.

### Light responses of cortical neurons in vivo

**Fig. 7:**
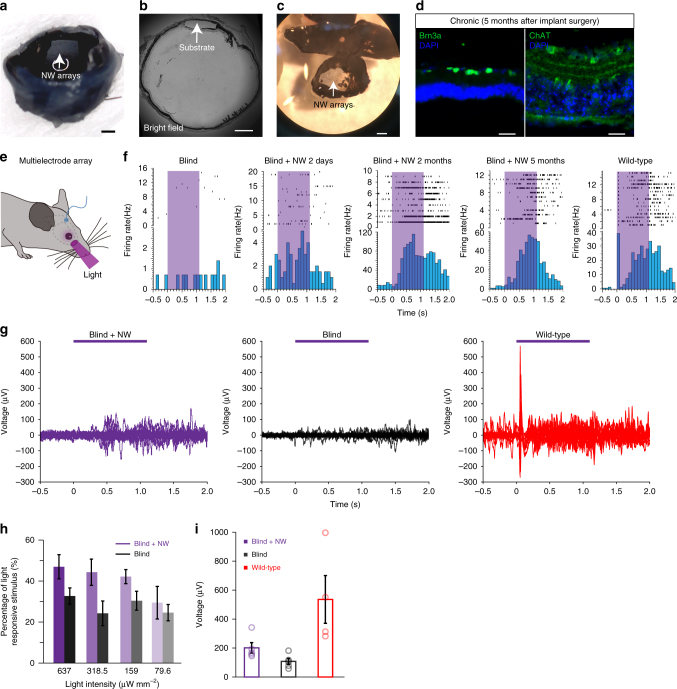
In vivo light responses after subretinal implant of NW arrays. **a** Explanted eye showing the position of the implanted NW arrays. **b** Slice of eyeballs in blind mice 7 weeks after implant surgery. **c** Dissected NW arrays-implanted eyecup 5 months after NW arrays implant surgery. **d** Brn3a and ChAT staining in higher magnification images showing the lamination of the NW arrays-implanted blind retina 5 months after implant. **e** Schematics of in vivo electrophysiology recording. The NW array was implanted subretinally, and multi-electrode array was inserted into the primary visual cortex to record light responses. **f** Raster plots and post stimulus time histograms (PSTHs) of spikes from V1 neurons in blind mice (left), NW arrays-implanted blind mice 2 days after implant (middle left), 2 months after implant (middle), 5 months after implant (middle right), and wild-type mice (right). The purple shade area indicates the presentation of light. **g** 15 traces of near UV light-evoked LFP responses in V1. Purple line indicates the presentation of light. **h** Percentage of stimuli that elicited responses in blind mice and NW arrays-implanted blind mice using different light intensities (Blind + NW: 637 μW mm^–2^, *n* = 6; 318.5 μW mm^–2^, *n* = 5; 159 μW mm^–2^, *n* = 5; 79.6 μW mm^–2^, *n* = 5. Blind: 637 μW mm^–2^, *n* = 6; 318.5 μW mm^–2^, *n* = 4; 159 μW mm^–2^, *n* = 4; 79.6 μW mm^–2^, *n* = 4. Two-tailed Student’s *t*-test, *P*_637_ = 0.016). **i** VEP amplitude in NW arrays-implanted blind mice, blind mice, and wild-type mice (*n* = 5 for NW arrays-implanted blind mice, *n* = 5 for blind mice, *n* = 4 for wild-type mice. Two-sided Wilcoxon rank-sum test, *P*_blind vs. blind+NW_ = 0.056). Data are presented as mean and S.E.M. Scale bars: 500 μm (**a**–**c**), 20 μm (**d**)

To further exhibit the potential utilization of NW arrays in retinal prosthesis, we conducted subretinal implant surgery of NW arrays in blind mice. Figure 7a–c showed the substrate of NW arrays implanted into the subretinal space. 5 months after the implant surgery, the lamination of the blind retina, as well as the RGCs labeled by Brn3a was unaffected by the implant (Fig. 7d). We presented near UV light to one eye of the blind mice and recorded the light-evoked spikes and visually evoked potentials (VEPs) in the contralateral primary visual cortex (Fig. 7e). In the blind mice, no obvious light-evoked spikes were detected (Fig. 7f, left column). UV light elicited robust spiking responses in all implanted blind mice 2 days after implantation (Fig. 7f, middle left column) and wild-type mice (Fig. 7f, right column). In addition, we conducted recordings in chronically implanted mice of both 2 months and 5 months after the implant surgery. The light evoked spiking activities were more prominent in chronically implanted mice (Fig. 7f, middle and middle right columns). UV light also triggered robust local field potentials (LFP) changes in the implanted blind mice (Fig. 7g). The fraction of stimuli that elicited LFP responses for blind mice remained the same for different light intensities, whereas that for the NW arrays-implanted blind mice increased with increasing light intensity (Fig. 7h), confirming the clear light response restoration of blind mouse retinas in vivo. The VEP amplitude in NW arrays-implanted blind mice was larger than that in blind mice, but still smaller than that in wild-type mice (Fig. 7i). We also observed light evoked spiking activities in the superior collicus (SC) in NW arrays-implanted blind mice (Supplementary Fig. 11).

The vision in near UV was investigated in vivo in alive *rd1*/*cDTA* mice. Although cornea, lens and other tissues in the eye absorb some UV light, mouse retinas can still be sensitive to UV light^36^. The absorption of UV light (375/28 nm) in the cornea and lens of the mouse was estimated to be ~42%. The near UV light responses in V1 in anaesthetized wild-type mice were measured in vivo (Supplementary Fig. 12a). It is clear that near UV light triggered both LFP and spiking activities across V1 (Supplementary Fig. 12b, c), indicating that wild-type mice have vision in UV^37^.

### Recovery of the light sensitivity in awake-behaving mice

**Fig. 8:**
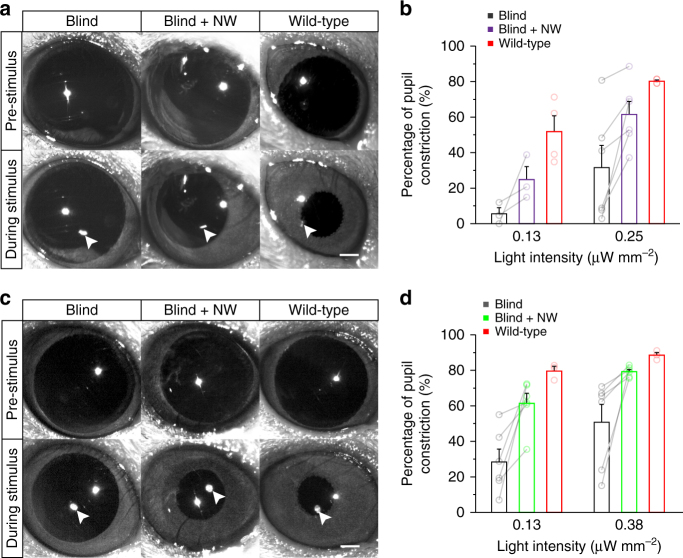
Pupillary light reflex 4–8 weeks after subretinal implant of NW arrays. **a** Infrared images of the left (with no implant) and right (implanted with NW arrays) eyes of the same blind mice, as well as the left eyes of wild-type mice immediately prior to light stimulus and maximum pupil constriction during near UV light stimulus. The left eye served as blind control. White arrows indicate the reflections of the UV LED on the cornea of the eye. **b** Pupil constrictions of blind eyes, NW arrays implanted blind eyes (one eye implanted with NW arrays and the contralateral eye as blind control), and wild-type eyes under different LED irradiances with wavelength peaked at 395 nm. 0.13 μW mm^–2^: *n* = 3 for blind mice, *n* = 4 for wild-type mice. 0.25 μW mm^–2^: *n* = 6 for blind mice, *n* = 4 for wild-type mice (two-tailed paired Student’s *t*-test, *P*_blind vs. blind+NW_ = 0.020). **c** Infrared images of the left (with no implant) and right (implanted with NW arrays) eyes of blind mice, as well as the left eyes of wild-type mice immediately prior to light stimulus and maximum pupil constriction during green light stimulus. The left eye served as blind control. White arrows indicated the reflections of the green LED on the cornea of the eye. **d** Pupil constrictions of blind eyes, NW arrays implanted blind eyes (one eye implanted with NW arrays and the contralateral eye as blind control) and wild-type eyes under different LED irradiances with wavelength peaked at 580 nm. *n* = 6 for blind mice, *n* = 3 for wild-type mice (0.13 μW mm^-2^: two-tailed paired Student’s *t*-test, *P*_blind vs. blind+NW_ = 0.014; 0.38 μW mm^-2^: two-sided Wilcoxon signed-rank test, *P*_blind vs. blind+NW_ = 0.031). Data are presented as mean and S.E.M. Scale bars: 500 μm (**a**, **c**)

In blind mice, the light-induced pupil constriction was largely impaired due to the loss of photoreceptors in the retina (Fig. 8a). Many retinal prosthesis studies use PLR to confirm the behavioral recovery of light sensitivity^34,38–40^. We implanted NW arrays subretinally into one eye of blind mice (Supplementary Fig. 13). The PLR to near UV light in the implanted eye was improved compared to that in the contralateral (control) eye at both 0.13 μW mm^–2^ and 0.25 µW mm^–2^ 4–8 weeks after implant surgery (Fig. 8b, Supplementary Movie 2 in the Supporting Information). Likewise, the PLR to green light in the implanted eye was also improved at both 0.13 μW mm^–2^ and 0.38 μW mm^–2^ (Fig. 8c, d, Supplementary Movie 3 in the Supporting Information). Some of the implanted eyes reached the same level of PLR as that in wild-type mice, indicating the recovery of light sensitivity in multiple colors.

## Discussion

The rational design and fabrication of photoresponsive artificial photoreceptors are important for developing advanced retinal prosthetic devices. Several possible mechanisms including photovoltaic and photothermal stimulations have been investigated^14^. Although it was recently reported that the photothermal effect by direct infrared neural stimulation is an alternative for retina prosthesis^41^, it requires the use of photoabsorbers in close proximity of the target cells^42^. The Au nanoparticles synthesized in our work have diameters of ~10 nm and are strongly bound to TiO_2_ nanowire surfaces. Although the photo-thermal conversion efficiency of small Au nanoparticles is high^43^, according to theoretical calculations, the temperature increase at the surface of Au nanoparticles in water is substantially lower for Au nanoparticles with diameters of 10 nm than those with diameters of 50 nm^44^. Thus, the photothermal stimulation of RGCs from our 10-nm-diameter Au nanoparticles should be minor. Instead, the Au nanoparticles in our work enable efficient charge injection into TiO_2_ nanowires upon photo-illumination. From our experimental data, it is clear that the RGC responses are closely correlated to the photocurrent stimulation of the Au-TiO_2_ nanowire systems. In addition, the major responses from the UV regime confirm the photovoltaic contribution of TiO_2_ nanowires for RGC stimulation. Thus, the mechanism reported in this work is mainly attributed to photovoltaic stimulation.

It is noted that the estimated receptive field was ~300 µm using 45 μm light spot. However, there was an increase in responsive percentage of RGCs when the size of the stimulation light spot was increased from 100 µm to 600 µm. The reason for the increase in RGC response for larger light spot is due to the increased photovoltaic current induced by larger light spot (Supplementary Fig. 9a, b). On the other hand, photocurrent induced by 45 μm light spot that was 300 µm away was too small to induce RGC responses.

Compared to other materials, the 1D oriented NW arrays mimic the morphology of rod and cone photoreceptors, and offer several distinctive features. First, the unique 1D anisotropic morphology of NW arrays provides an efficient photo-to-electric conversion modality, similar to the NW arrays-based photovoltaic devices. The nanoscale length of the NW radial dimension allows for fast separation of photogenerated carriers^45^. Meanwhile, the micron-scale length of the NW axial dimension can efficiently absorb incident light with excellent photoabsorption coefficients, with a thickness much smaller than normal photoreceptor cell layers. As shown in the pupillary light reflex experiment, the sensitivity of blind mice implanted with NW arrays was similar to that in wild-type mice.

Second, the vertically oriented NW arrays are densely and uniformly grown over the surface, which resemble photoreceptor rods and cones in the outer retina layer. The space between neighboring NW arrays is on the order of hundreds of nanometers, and no electrodes or power supplies are needed, thus allowing for a much higher spatial density (~10 NW arrays per μm^2^) of active stimulation elements than most of the previously reported photoresponsive surface. The calcium imaging experiments in our work demonstrate that adjacent neurons can be activated simultaneously by NW arrays. In addition, as shown in our work, the feature size of light response can be as small as ~50 and ~300 μm for near UV and green light, respectively. Further increase in the sensitivity of individual NW arrays may continue to improve the spatial resolution.

Third, the rough morphologies of the vertically oriented NW arrays are beneficial for direct interfacing with cultured neurons^46^, retinal tissues or in vivo subretinal implant into live mice, with good biocompatibility and photo/chemical stability for over 8 weeks. The interface between the vertically oriented NW arrays and retinal cells/tissues leads to efficient light information processing. Our in vivo experiments have suggested the possibility of using NW arrays for chronic implant.

Fourth, although NW arrays used in present work have not been capable for color vision, further development of multiple nanowire types with different spectral responses may be a potentially feasible strategy to realize such a scenario in the near future. For example, color vision can become possible if 1) type A nanowire is sensitive to red color and type B nanowire is sensitive to green color; 2) type A nanowire induces smaller firing rate and type B nanowire induces larger firing rate in RGC; 3) after training, the visual cortex can rewire to recognize the difference in RGC activities, and hence differentiate red color from green color.

In summary, we demonstrated a vertically oriented Au–TiO_2_ NW arrays as artificial photoreceptors, which absorbs light and generates photovoltage, and consequently triggers spiking activities in the interfaced neurons and restore light responses in the photoreceptor-degenerated retina. The spatial resolution is approaching or comparable to ~50 μm, and the size of receptive field is comparable to that in wild-type mice. The light-response inhibition by glutamatergic antagonists suggests the NW arrays-interfaced retinas were capable of processing visual information through innate retinal circuits. Moreover, functional and behavioral restoration of light sensitivity has been demonstrated with subretinal implant of the Au–TiO_2_ NW arrays in blind mice, suggesting the possibility for using NW arrays as prosthetic devices.

## Methods

### Synthesis of TiO_2_ and Au–TiO_2_ nanowire arrays

TiO_2_ NW arrays were vertically grown on fluorine-doped tin oxide (FTO)-coated glass (Wuhan Ge-Ao Ltd., China) or polydimethylsiloxane (PDMS) substrates by a hydrothermal method, in which tetrabutyl titanate (TBOT) was used for the Ti precursor. In a typical synthesis, 0.1 g of Ti foil and 18 mL of 0.1 M HCl solution were put into a Teflon-lined stainless steel autoclave with a total volume of 25 mL. The PDMS elastomer was then immersed into the solution after ultrasonic cleaning for 30 min in a 1:1:1 (v/v/v) mixture of acetone, ethanol and deionized (DI) water. The hydrothermal synthesis was conducted at 150 °C for 12 h in an electric oven. For the Au-TiO_2_ NW arrays synthesis, after the fabrication of TiO_2_ NW arrays, Au nanoparticles were deposited on TiO_2_ NW arrays by a solution reduction approach. Briefly, an FTO substrate with TiO_2_ NW arrays was soaked into 0.01 M HAuCl_4_ (with pH tuned to 4.5 by adding 0.2 M NaOH) for 2 h. The sample was then thoroughly washed by DI water, dried in air and annealed in air at 300 °C for 2 h. For in vivo implantation and measurement, after nanowire synthesis, the side of FTO substrate grown with TiO_2_ nanowires was pre-covered with a thin layer of polymethylmethacrylate (PMMA) and incubated at 85 °C for 5 min. Then, the FTO substrates were etched in a hydrogen fluoride solution (20%) for ~9 h to reduce the thickness of FTO substrates to below 100 μm, followed by removal of PMMA in aceton.

### Photoelectric measurement

The photoelectric measurement was carried out using a CHI 660D electrochemical workstation (CH Instruments, Inc., USA) with a 3-electrode system, in which the Au–TiO_2_ NW arrays photoanode, a coiled Pt wire and an Ag/AgCl were used as the working, counter and reference electrodes, respectively. The photoanode was illuminated under 100 mW cm^–2^ of simulated sunlight, using a solar simulator equipped with a 150 W xenon lamp and an AM 1.5 G filter (94022A, Newport Inc., USA). A phosphate buffer saline (PBS, pH 7.4) was used as the electrolytes. Different filters were applied to obtain UV (375/28 nm, full intensity 133 μW mm^–2^), blue (470/20 nm, full intensity 691 μW mm^–2^) and green light (546/12 nm, full intensity 470 μW mm^–2^).

### Animals and genotyping

All procedures were conducted in accordance with the guidelines of the Institutional Animal Care and Use Committee at Shanghai Public Health Clinical Center. Wild-type (C57BL/6J) mice were obtained from the Slac Laboratory Animal Co. (Shanghai, China). All animals were raised and bred at 25°C, 50% relative humidity, 12 hours light and dark cycles. For sorting the *cl* positive and *rd1* homozygote mice, two pairs of primers and a restriction enzyme (ThermoFisher, HpyF3I (DdeI)) which recognizes C^TNAG sites were used. To differentiate *rd1*^−/−^ and *rd1*^-/+^, primers 5′-CATCCCACCTGAGCTCACAGAAAG-3′ and 5′-GCCTACAACAGAGGAGCTTCTAGC-3′ were used. After digestion, homozygote mice had 2 mutant bands: 126 bp and 155 bp. Heterozygous mice had two mutant bands and a wild-type band. Mutant bands at 126 bp and wild-type band at 301 bp. To distinguish *cl* (also called *cone-DTA*) knockout and wild-type, primers 5′-CAAGGAAATTATGACGATGATTGG-3′ and 5′-GGCTTGAGCCATATACTCATACATCGC-3′ were used with mutant band at about 450 bp. Mice used in experiments were either male or female and from multiple litters. All the mice were more than 7 weeks old, and more specific ages could be seen in the corresponding sections of Methods. Sample size was chosen to be larger than 5 for statistical analysis. In some cases, due to the limited number of available mice, sample size was 4, and no statistical analysis was conducted. Since our experiments were designed to look for all-or-none response, we did not randomize our samples. No specific blinding was done in the in vitro and in vivo electrophysiology recordings and Ca^2+^ imaging experiments since all data analysis was conducted using automated methods. Blinding was conducted between PLR experiment and data analysis.

### Retinal tissue preparation

Mice were anesthetized with 10% chloral hydrate (0.3 mL per 100 g weight). One eye was enucleated and rapidly placed in Ringer’s solution consisting of (in mM) NaCl 124, KCl 2.5, CaCl_2_ 2, MgCl_2_ 2, NaH_2_PO_4_ 1.25, NaHCO_3_ 26 and glucose 22, pH 7.35 and oxygenated with 95% O_2_ and 5% CO_2_. Then the retina was dissected and placed on a filter paper (MerckMillipore, Germany) in the recording chamber.

### Photo-stimulation

Light stimulation was performed on a setup consisting of a Zeiss upright DIC microscope (Examiner A1, Zeiss, Germany). Near UV, blue and green light were generated from the mercury lamp (X-Cite 120, Lumen Dynamics, USA), filtered by fluorescence cubes (UV: 375/28 nm; Green: 546/12 nm; Blue: 470/20 nm, Zeiss Inc., Germany) and provided through the ×40 water-immersion objective. A circular illumination spot around neurons was obtained by passing light through a pin hole and focusing the light with a water immersion ×40 objective.

### Patch-clamp recording of retinal ganglion cells

Action potentials were recorded using MultiClamp 700B patch-clamp amplifier (Molecular Devices, USA), and digitized by Digidata 1440 (Molecular Devices, USA) under DIC microscope (Zeiss, Germany) at room temperature. A glass pipette (5–10 MΩ) was pulled by P-97 micropipette puller (Sutter Instruments, USA) filled with internal solution (in mM, potassium gluconate 105, KCl 5, CaCl_2_ 0.5, MgCl_2_ 2, EGTA 5, HEPES 10, Mg-ATP 4, GTP-Na 0.5, sodium phosphocreatine 7, Lucifer Yellow 0.05%, PH 7.4). pClamp 10 (Molecular Devices, USA) was used for data analysis. The investigator was blinded to the group allocation when analyzing the light-response of RGCs but not during the patch-clamp experiment. Dendritic arbors of RGCs were labeled by filling the patch pipette with Lucifer Yellow and imaged after the whole-cell patch clamp experiment. The distance *d* from the tip of the longest arbor to the center of the cell body was measured. The size of the dendritic arborizations was calculated by π*d*^2^. The concentrations for glutamate receptor antagonists were: L-AP4: 50 µM, D-AP5: 50 µM, NBQX: 20 µM.

### Intravitreous injection and Ca^2+^ imaging in vitro

The mice were anesthetized by 2% chloral hydrate (0.2 mL per 10 g weight). 1 μL AAV-syn-GCaMP6s virus were injected into the retina of *rd1*/*cDTA* knockout mice at P28 using NanoJectII (Drummond scientific company, Germany). Expression of GCaMP6s in the retina started 2 weeks after the injection surgery. After 3-4 weeks, expression reached the optimal level and retinas were dissected for imaging experiments. A series of fluorescent images were captured and collected at 10 Hz (512 × 512 pixels) by Flash 4.0 (Hamamatsu, Japan) at room temperature, and were analyzed by ImageJ software. ∆*F*/*F*_0_ was calculated as (*F*–*F*_0_)/*F*_0_. *F*_0_ was equal to the mean of fluorescence signals from the first 5 frames.

### Immunohistochemistry

Retina was fixed in 4% PFA for 5−7 h at 4 °C immediately after dissection. For retina slice immunohistochemistry, 10% (30 min, room temperature), 20% (30 min, room temperature) and 30% (overnight, 4 °C) sucrose were used to dehydrate the fixed retina. The retina was embedded in OCT compound (Sakura) and stored at −80 °C for more than 2 h. Fourteen-micrometer slices were cut (Leica CM 1950, Leica, Germany) and washed 3 times for 15 min with 0.05 M TBS to wash away the OCT. After immersing slices in 0.5% Triton-X-100 for 20 min, slices were incubated in a blocking solution consisting 10% Donkey serum (Jackson Immunoresearch, USA), 1% bovine serum albumin (BSA), and 0.05% Triton-X-100 in 0.05 M TBS (DST) for 2 h at room temperature. Then slices were immediately covered by the primary antibody (ANTI-Choline Acetyltransferase antibody^47^, MILLIPORE (AB144P), 1:200) solution and hybridization for 20–24 h at 4 °C. The slices were then washed 6 times for 10 min in TBS to get rid of the primary antibody, followed by incubated with secondary antibody (Donkey anti-Goat conjugated to Alexa Flour 594, 1:300, Jackson ImmunoResearch, USA) at room temperature for 2 h in dark. After removing the secondary antibody and washing 3 times for 10 min, the slices were stained in 1:3000 DAPI solution for 10 min and washed 3 times for 10 min. Slices were air dried and mounted. Fluorescent images were taken under epifluorescence microscope (Olympus, Japan).

The immunohistochemistry protocol for whole-mount retinas was similar to that in the retinal slices. After patch-clamp recording, retinas were fixed in 4% PFA for 5−7 h at 4 °C, washed by TBS, incubated in 0.5% Triton-X-100 for 30 min, blocked by 10% DST for 3 h at room temperature. The retina was then transferred into the primary antibody (ANTI-GFP antibody^48^, Avēs Labs Inc. (GFP-1020), 1:1000; ANTI-Brn3a antibody^49^, Santa Cruz Biotechnology (SC-31984), 1:500; ANTI-Lucifer Yellow antibody^50^ Invitrogen (A5750), 1:500) solution and incubated for 24−30 h at 4 °C. After washing in TBS, retinas were transferred to the secondary antibody (Donkey anti-Chicken conjugated to Alexa Flour 488, 1:300, Jackson ImmunoResearch, USA; Donkey anti-Goat conjugated to Alexa Flour 594, 1:300, Jackson ImmunoResearch, USA; Donkey anti-Rabbit conjugated to Alexa Flour 488, 1:300, Jackson ImmunoResearch, USA) solution for 3 h at room temperature in the dark. After the secondary antibody was washed off, whole-mounted retinas were air dried and mounted by Fluruomount-G (Southern Biotech, USA). Fluorescent images were obtained by fluorescence imaging microscope (Eclipse Ni, Nikon Inc, Japan) and confocal multi-photon scanning microscope (AIR-MP, Nikon Inc, Japan) and analyzed in ImageJ software 1.48v (NIH) and NIS-Elements AR software ver. 4.30.01 (Nikon Inc).

### Surgery for in vivo electrophysiology recording

Mice aged at P56 were deeply anaesthetized with 2.5% isoflurane in oxygen for 20 min before the surgery. The nose and mouth of the mice were placed in a respiratory mask infused with 0.5−1% isoflurane. The mice were kept on a heating pad (FHC Inc., USA), followed by subcutaneous injection of 1% lidocaine (10 mg mL^–1^ lidocaine in saline; MP Biomedicals, USA) under the scalp. After removal of the scalp, the front and back of the skull were glued onto two copper rods and assembled onto a rotatable mounting platform. A craniotomy window in the skull was created stereotaxically in V1 (3.1−4.2 mm posterior to bregma and 2.0−3.2 mm lateral, at a depth of 300–500 μm), and the dura was removed carefully. The craniotomy was filled with warm (37 °C) sterile buffered saline (150 mM NaCl, 2.5 mM KCl, 10 mM HEPES, pH 7.4) throughout the experiment. The recording platform was set up on a rotatable base (Thorlabs Inc., USA). The platform was adjusted such that the craniotomy plane was vertical to the electrodes.

### Visual stimuli and in vivo electrophysiology recordings

Isoflurane anesthesia was adjusted to 0.5% to maintain a stable respiratory rate. Visual stimuli were consisted of 1 s UV light with various intensities followed by 59 s non-stimulation period. The whole-eye UV stimulus were produced by mercury lamp at various intensities and last for 1.06 s. For multielectrode array recordings, neuronal signals were recorded in V1 using a 4 × 4 microelectrode array interfaced with Power 1401 (Cambridge Electronic Design, UK). Platinum/Iridium (70%/30%) opto-MEA (1 MΩ, 250 μm apart, Microprobes, USA) was inserted into L2/3 of primary visual cortex. Electrical signals were amplified (Microelectrode AC Amplifier 1800, A-M Systems, Inc., USA), high-pass filtered at 1 Hz and sampled at 10 kHz using Power 1401. Spike activity was high pass filtered at 300 Hz. We used 5 times the number of standard deviation (sigmas) in the mean of the peak height histogram to threshold the spike signals. Multi-unit spikes were initially clustered using K-means in Offline sorter (Plexon Inc., USA) and manually grouped similar clusters if necessary. Firing rates were calculated in Neuroexplorer (Plexon Inc., USA) and peri-stimulus time histograms (PSTHs) were plotted in Spike2 software (Cambridge Electronic Design, UK).

### Implant surgery

Eight to ten weeks old *rd1*/*cDTA* (blind mice) were anaesthetized. Up and down eyelids were suture to fix the eye-ball in proper position which is suitable for implantation. The mice were then fixed on the stereotaxic aparatus. A small cut was made in the sclera, 1–2 μL of 0.9% sodium chloride solution was injected quickly and carefully by nano-ject (Drummond Instrument) to leave enough space for NW arrays implantation. After that, an expansion cut was quickly made at about 45–60 angle to the nasal-temporal axis. The size of the cut was determined by the size of the substrate for NW arrays. The typical area of the implanted NW arrays is 0.5 mm^2^–1.5 mm^2^. The substrate was held by a sharp tweezer and inserted into the cut under microscope. After the implantation, wash the eye-all by 0.9% sodium chloride solution and remove the suture to retract the eye-ball. Mice were put back to the home cage to recover.

### Retinal tissue preparation for scanning electron microscope

Eye-balls were extracted about 5 days after NW arrays implantation. The lens was removed and the remaining eye-cup was fixed in 4% PFA at 4 °C overnight, dehydrated in 30%, 50%, 70%, 90%, 95%, and 100% ethanol for 1 h in each solution, and air-dried at room temperature. The retina was trimmed to reveal the edge of the tissue before SEM.

### Pupillary light reflex

Metal plates were implanted onto the skull of mice prior to behavioral experiments. Following 2 hours of dark adaption, unanaesthetized mice (over 50 days old) were head-fixed through metal plates under infrared illumination. Light stimuli were provided by an ultra violet LED (peaked at 395 nm), as well as green LED (peaked at around 560 nm), to one eye, while a near-infrared camera (JAI, Denmark) recorded the response from the same eye. Light intensities were measured at the cornea using an optical power meter (Thorlabs, USA). Each session was recorded at 62 Hz frame rate for 1 minute, starting 5 s before a 20 s light stimulus. Pupil areas were measured using ImageJ (NIH, USA). Percentage of pupil constrictions was calculated as $\left({{\mathrm{1 - }}\frac{{a_{{\mathrm{min}}}}}{{a_0}}} \right){\mathrm{ \times 100}}\%$ ($a_{{\mathrm{min}}}$: minimum pupil area during light exposure; $a_0$: pupil area immediately prior to light exposure). One mouse was excluded from pupillary light reflex analysis due to frequent spontaneous pupillary constriction which disrupted the judge of light reflex.

### Statistics

All data are represented as mean and S.E.M. The differences were tested by OriginPro software ver. 9.0.0 (Hewlett–Packard Company) and SPSS software version 23.0 (IBM). All comparisons were made by unpaired two-tailed Student’s *t*-tests, two-sided Wilcoxon rank-sum tests and Chi-square tests, except for the PLR experiments where paired two-tailed Student’s *t*-tests or two-sided Wilcoxon signed-rank tests were used. All data met the assumptions of the test for distribution with similar variance statistically compared. *P* values <0.05 were considered significant.

### Data availability

The data that support the findings of this study are available from the corresponding author upon reasonable request.

## Electronic supplementary material

Supplementary Information

Description of Additional Supplementary Files

Supplementary Movie 1

Supplementary Movie 2

Supplementary Movie 3
