# Supplementary material for: Nanowire arrays restore vision in blind mice
Source: Nat Commun. 2018 Mar 6;9:786. doi: 10.1038/s41467-018-03212-0 (PMC5840349; doi:10.1038/s41467-018-03212-0)
Supplement: Supplementary file 1 — Supplementary Information [file 41467_2018_3212_MOESM1_ESM.pdf]

Supporting Information for

Nanowire Arrays Restore Vision in Blind Mice

Jing Tang,[1,†] Nan Qin,[1,†] Yan Chong,[1,†] Yupu Diao[1], Yiliguma[1], Zhexuan Wang[1], Tian Xue[2], Min Jiang,[1] Jiayi Zhang,[1,*] Gengfeng Zheng[1,*]

[1]Laboratory of Advanced Materials, Institutes of Brain Science, State Key Laboratory of Medical Neurobiology, Shanghai Key Laboratory of Molecular Catalysis and Innovative Materials, Department of Ophthalmology, Zhongshan Hospital, Fudan University, Shanghai, China

[2]School of Life Sciences and Hefei National Laboratory for Physical Sciences at Microscale, University of Science and Technology of China, Hefei, China.

*Corresponding authors: Gengfeng Zheng, Jiayi Zhang

Emails: gfzheng@fudan.edu.cn (G.Z.); jiayizhang@fudan.edu.cn (J.Z.).

[†] J.T., N.Q. and Y.C. contributed equally to this work.

Supporting Figures

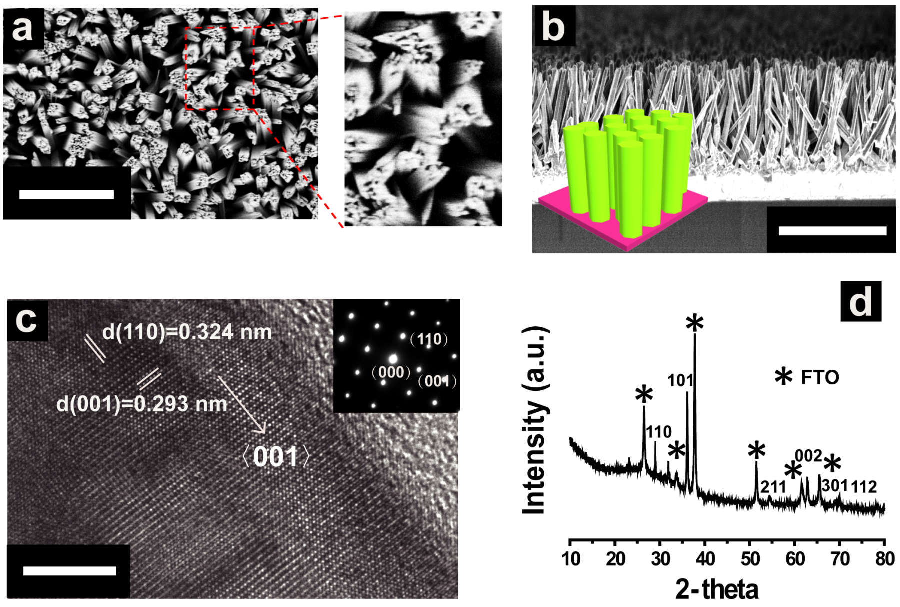

Supplementary Figure 1 | (a) Top-view and **(b)** side-view SEM images of TiO_2 NW arrays on an FTO substrate. **(c)** HRTEM image and (inset) selected area electron diffraction pattern of a representative single-crystalline TiO_2 NW. **(d)** XRD pattern of TiO_2 NWs on an FTO substrate. The peaks of FTO are indicated by asterisks. Scale bars: 1 μm (**a**), 2 μm (**b**), 5 nm (**c**).

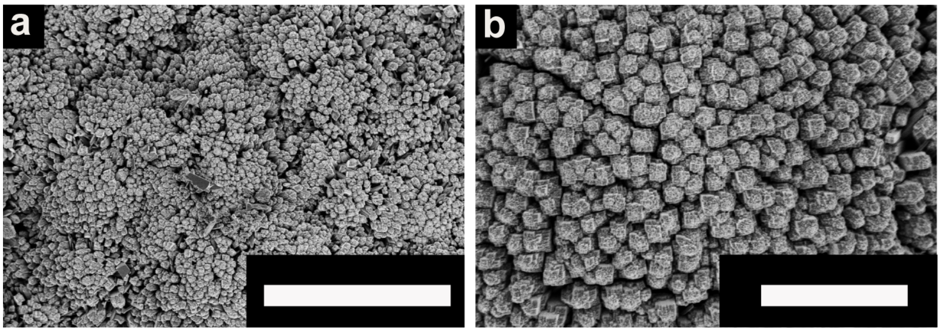

Supplementary Figure 2 | (**a, b**) Top-view SEM images of Au nanoparticle-decorated TiO_2 nanowire arrays on the PDMS. Scale bars: 10 μm (**a**), 2 μm (**b**).

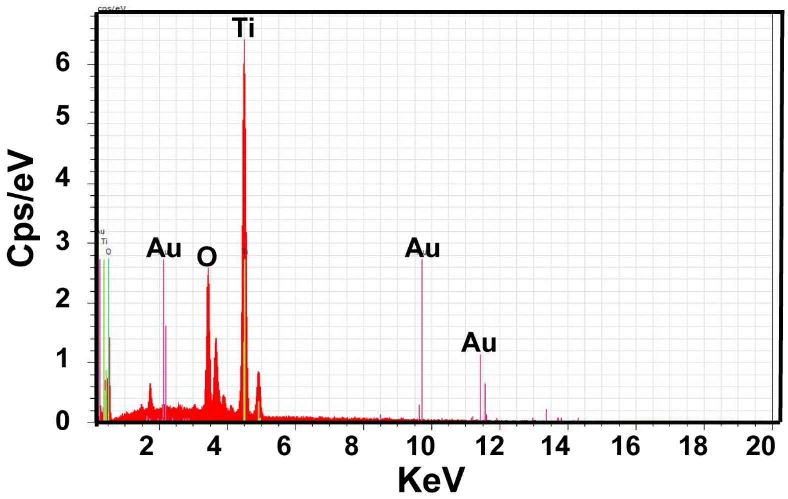

Supplementary Figure 3 | EDX spectrum of the Au-TiO_2 NWs. The molar percentages of Au and Ti were calculated as ~ 0.11% and 71.77%, respectively.

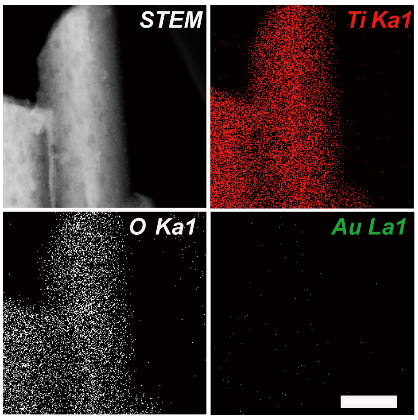

Supplementary Figure 4 | Scanning TEM image and corresponding EDX elemental mapping data of Au-TiO_2 NWs. Scale bar: 100 nm.

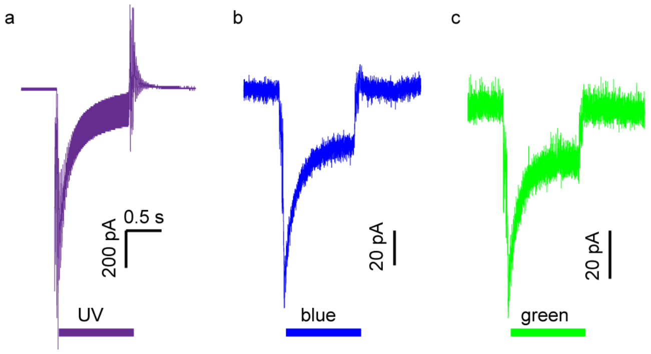

Supplementary Figure 5 | Photocurrent versus time from an Au-TiO_2 NW array generated by illumination of UV (a) , blue (b) and green (c) light, respectively. Different filters were applied to obtain UV (375/28 nm, 133 μW mm^{-2}), blue (470/20 nm, 691 μW mm^{-2}) and green light (546/12 nm, 470 μW mm^{-2}).

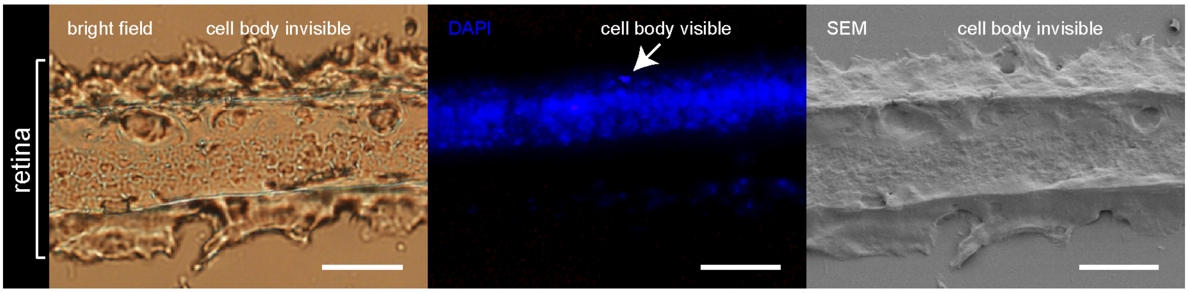

Supplementary Figure 6 | Bright light, DAPI fluorescence and Scanning Electron Microscope images of the same 30 µm retinal slice. Scale bars: 50 µm.

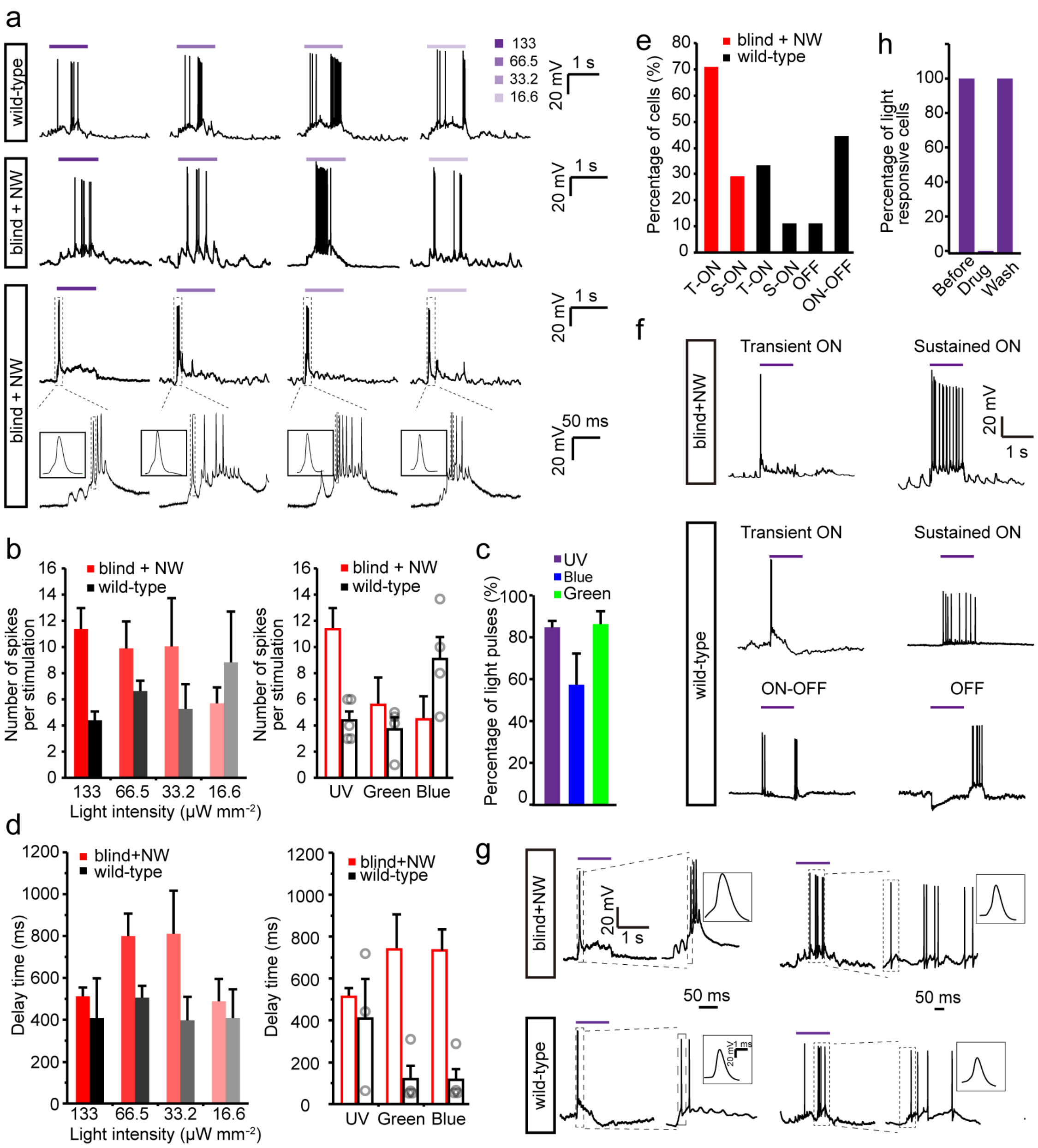

Supplementary Figure 7 | Light responses in NW array-interfaced blind mouse retinas. (a) RGC responses to UV light with different light intensities. The light intensity values are shown with units of μW mm^{-2}. Purple lines represent the presence of UV light. The bottom panel shows zoom-in spike activities. **(b)** Number of spikes per stimulation. Blind + NW: n = 43, 23, 13, 17 RGCs in 23, 17, 11, 10 retinas for 133, 66.5, 33.2, 16.6 μW mm^{-2}, respectively; Green, n = 8 RGCs, 5 retinas; Blue, n = 5 RGCs, 4 retinas. Wild-type: n = 5, 5, 7, 6 RGCs in 4, 4, 6, 4 retinas for 133, 66.5, 33.2, 16.6 μW mm^{-2}, respectively; Green, n = 4 RGCs, 2 retinas; Blue, n = 4 RGCs, 3 retinas. **(c)** Percentage of light pulses triggering spiking activities in all light-responsive RGCs for UV (n = 65

RGCs, 25 retinas), blue (n = 5 RGCs, 4 retinas) and green (n = 9 RGCs, 6 retinas) light in NW array-interfaced blind retinas. **(d)** Latency to the onset of near UV, green, blue light. Blind + NW: n = 45, 22, 13, 18 RGCs in 24, 17, 11, 10 retinas for 133, 66.5, 33.2, 16.6 μW mm^{-2}, respectively; Green, n = 11 RGCs, 5 retinas; Blue, n = 5 RGCs, 4 retinas. Wild-type: n = 3, 3, 6, 6 RGCs in 3, 3, 3, 4 retinas for 133, 66.5, 33.2, 16.6 μW mm^{-2}, respectively; Green, n = 4 RGCs, 2 retinas; Blue, n = 4 RGCs, 3 retinas. **(e)** Percentages of RGCs with different light-response patterns and examples shown in **(f)**. Blind + NW: n = 86 RGCs, 25 retinas; Wild-type: n = 9 RGCs, 9 retinas. . **(g)** Spike details in wild-type and NW-interfaced blind retinas. **(h)** Percentage of responsive RGCs before, during and after application of glutamate receptor antagonist in light-responsive RGCs (n = 6 RGCs, 6 retinas). Data are presented as mean and S.E.M.

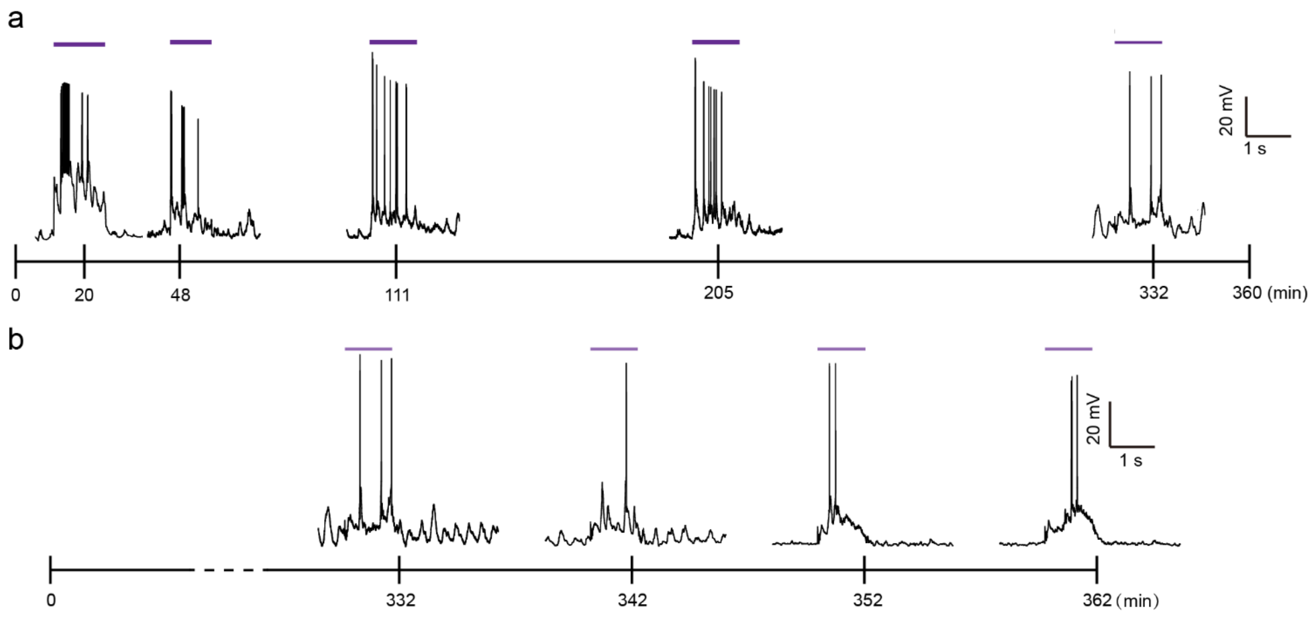

Supplementary Figure 8 | Time lapse responses in NW array interfaced retinas. (a) Light responses of RGCs *in vitro*. Time 0 represents the moment of putting the retina on top of the NW array. The first RGC was successfully patched 20 min later. **(b)** Light responses of one RGC *in vitro*. This RGC was successfully patched 332 min after putting the retina on top of the NW array.

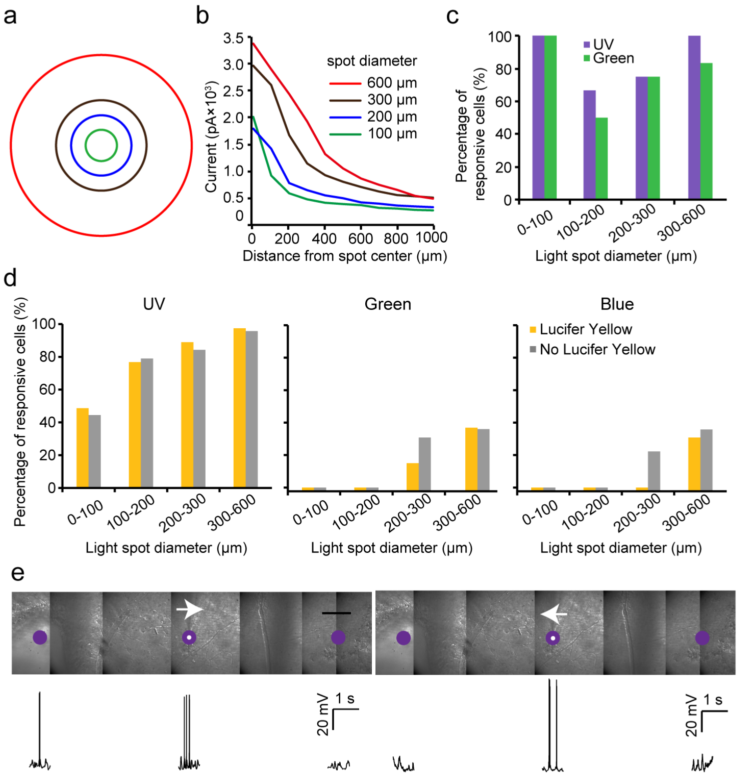

Supplementary Figure 9 | (a) Schematic for the light spot with different diameter. **(b)** The photocurrent of NW array triggered by different-size light spot measured in different distance from center of the spot. **(c)** Percentage of light-responsive cells with different light spot diameter in wild-type mice. UV: n = 9, 9, 8, 6 RGCs in 6, 6, 6, 5 retinas for 0 – 100, 100 – 200, 200 -300 and 300 – 600 µm, respectively. Green: n = 9, 8, 8, 6 RGCs in 6, 5, 6, 5 retinas for 0 – 100, 100 – 200, 200 -300 and 300 – 600 µm, respectively. **(d)** Percentage of light-responsive cells in response to different-size light spot with or without Lucifer Yellow (LY) in the internal solution. UV: LY, n = 37, 43, 27, 38 RGCs in 20, 21, 15, 20 retinas for 0 – 100, 100 – 200, 200 -300 and 300 – 600 µm, respectively. No-LY, n = 18, 19, 19, 23 RGCs in 13, 13, 13, 16 retinas for 0 – 100, 100 – 200, 200 -300 and 300 – 600 µm, respectively. Green: LY, n = 24, 19, 20, 38 RGCs in 14, 9, 11, 17 retinas for 0 – 100, 100 – 200, 200 -300 and 300 – 600 µm, respectively. No-LY, n = 13, 12, 13, 25 RGCs in 9, 9, 9, 14 retinas for 0 – 100, 100 – 200, 200 -300 and 300 – 600 µm, respectively. Blue: LY, n = 13, 11, 11, 13 RGCs in 6, 5, 6, 6 retinas for 0 – 100, 100 – 200, 200 -300 and 300 – 600 µm, respectively. No-LY, n = 8, 9, 9,

14 RGCs in 5, 6, 6, 8 retinas for 0 – 100, 100 – 200, 200 -300 and 300 – 600 μm, respectively. **(e)** The responses to moving UV light spots (purple circles, 55 μm) with two opposite directions. Arrows represent the moving direction of spots. White dots represent the location of the neuron. Top panel: DIC images of retina and three example locations of the light spot. Bottom panel: The corresponding light responses of different light spot locations. Average moving velocity: 0.0936 mm s^{-1} (left), 0.1674 mm s^{-1} (right). Scale bar: 100 μm (**e**).

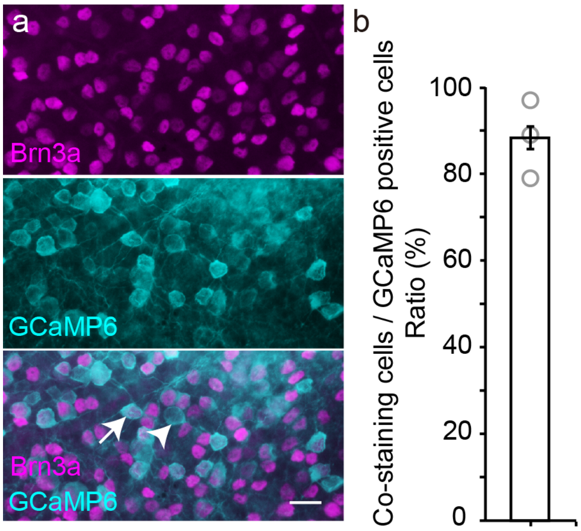

Supplementary Figure 10 | Most of the GCaMP6 positive cells are RGCs. (a) Immonuhistochemistry for Brn3a (up), GCaMP6 (middle), and merge of Brn3a and GCaMP6 (bottom) in the retina of blind mice. Arrowhead with long tail: co-staining of Brn3a and GCaMP6. Arrowhead with short tail: GCaMP6. **(b)** The ratio of Brn3a and GCaMP6 co-staining cells to GCaMP6 cells in the retina of blind mice (n = 3 areas from 1 retina). Data are presented as mean and S.E.M. Scale bar: 20 µm (**a**).

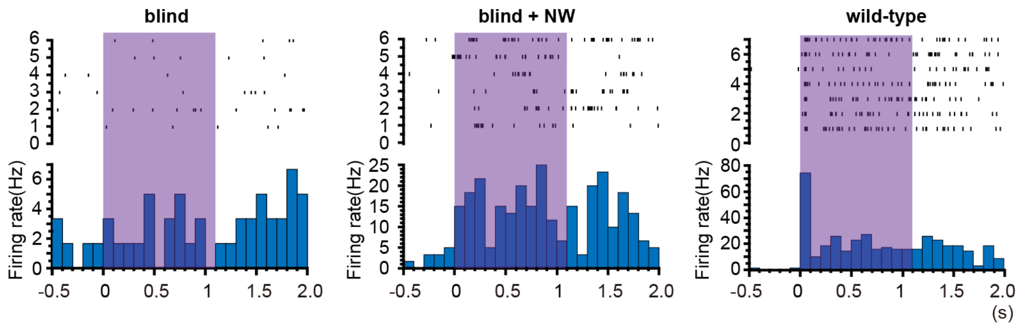

Supplementary Figure 11 | Raster plots and post stimulus time histogram (PSTH) of spikes from SC neurons. Left: blind mice, Middle: NW array-implanted blind mice 2 days after implant, Right: wild-type mice. The purple shade area indicates the presentation of light.

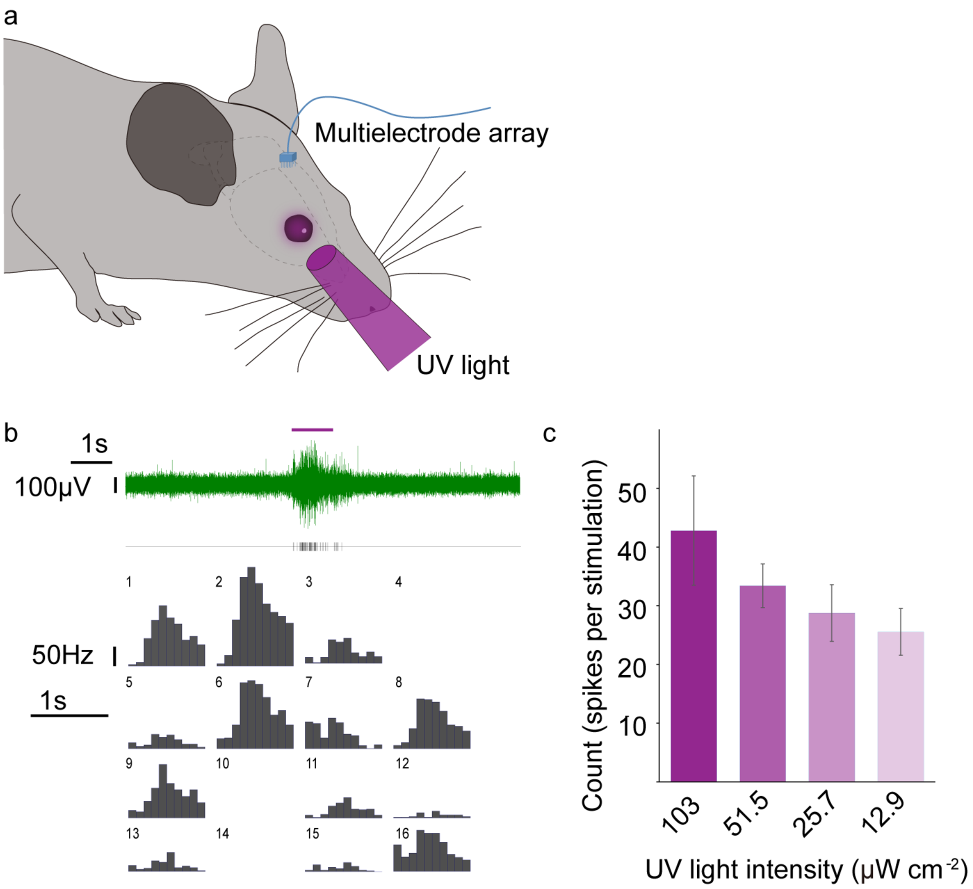

Supplementary Figure 12 | Response to UV light in primary visual cortex *in vivo* in anaesthetized wild-type mice (a) Schematic of the extracellular recording setup *in vivo*. UV light was presented in front of one eye through a light guide. Spiking activities in the contralateral primary visual cortex were recorded using a 16 channel multielectrode array. **(b)** Top: Green trace showed the high-pass filtered (>300 Hz) raw data recorded from one channel. Middle: Raster plot of spikes in the top trace. Each vertical black line represented one spike. Bottom: PSTH (post-stimulation time histogram) firing rate of neurons recorded from each channel. The numbers represented the channel. Each column represented average firing rate of the neuron within 100 ms. Channel 4 recorded the timing for the UV light stimulation. **(c)** Average number of spikes per 1 sec stimulation of all the neurons (n = 15) at different UV light intensity. Data are presented as mean and S.E.M.

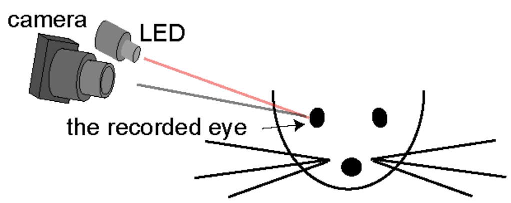

Supplementary Figure 13 | Schematic diagram of pupillary light reflex experiment.
