# Supplementary material for: Nanowire arrays restore vision in blind mice
Source: Nat Commun. 2018 Mar 6;9:786. doi: 10.1038/s41467-018-03212-0 (PMC5840349; doi:10.1038/s41467-018-03212-0)
Supplement: Supplementary file 2 — Description of Additional Supplementary Files [file 41467_2018_3212_MOESM2_ESM.pdf]

Description of Additional Supplementary Files

File Name: Supplementary Movie 1
Description: Calcium imaging of light responses in population retinal ganglion cells.

File Name: Supplementary Movie 2
Description: Pupillary light reflex to UV light in blind and wild-type mice.

File Name: Supplementary Movie 3
Description: Pupillary light reflex to green light in blind and wild-type mice.
